# Onychia Maligna of the Great Toe

**Published:** 1843-07-08

**Authors:** 


### ONYCHIA MALIGNA OF THE GREAT TOE.

BY A. COLLES, M. D.

I am of opinion, founded on some experience, that this disease may be cured without resorting to such a painful measure, [as excision of the diseased matrix,] by external applications alone, which are unattended with pain, and which, in a few days, will induce a considerable amendment; and even a perfect cure in the course of three or four weeks. The plan of treatment which I have pursued for some years, and, as yet, with invariable success, is as follows: I confine the patient to bed, and direct a poultice to the toe for two or three days; I then cleanse the ulcer carefully, by directing on it, from some height, a small stream of tepid water from a sponge; I next cut away as much of the loose nail as I can, without paining or irritating the sensitive surface around, and then I fumigate the part by means of the mercurial candle, containing ʒi. of the hydr. sulphuretum rubrum (olim cinnabar,) to two ounces of wax. This fumigation is to be applied night and morning, and after each, the toe should be gently enveloped in lint or linen, lightly spread with ung. spermaceti. In four or five days, the patient will express himself as considerably relieved; the discharge from the ulcer will be found of a healthy, purulent character, and the appearance of the whole part much more favourable. The fumigation is still to be persevered in, and all projecting portions of nail to be closely cut: I consider this latter direction as very essential, as thereby the mercurial fumes can have more free access to the surface of the ulcer. In proportion as the ulcer improves, it is interesting to observe, so does the condition of the growing nail; it acquires not only its natural firm and horny consistence, but also assumes its proper horizontal direction. For some time after the general surface of the ulcer has been healed, there still remain small spots of ulceration, generally at the angles, around some white germs of new nail; against these points the full force of the mercurial vapour should be directed; this can be effected by adding a small conical ivory tube to the funnel. I attribute much of the success of this treatment to the use of the mercurial candle, in preference to fumigation in the ordinary mode. I must observe, that during this course of treatment, the patient must absolutely abstain from walking, or even standing on the affected limb; exercise but for a single day will counterbalance all the amendment produced by a week's rest and fumigation. In the cases which I have thus treated, I have had no recourse to any constitutional treatment, or to any peculiar regimen; no doubt, cases must occur in which there will be derangement of the general health, and which will require suitable constitutional remedies, before we can expect to cure the local disease by mere local applications. I make no doubt that this ulceration may sometimes arise from, or be so intimately connected with some constitutional derangement, as to be incurable, without the aid of constitutional treatment. Mr. Wardrop, in his paper above alluded to, has recommended a cautious and judicious use of mercury, and has recorded some instances of its success.

The uniform success which has attended this plan of treating onychia, by mercurial fumigation, during the last few years I have adopted it, leads me to hope that the surgical operation I have already alluded to may in future be dispensed with; but even should more extended experience prove that I have been too sanguine as to this expectation, yet I feel persuaded that this practice cannot be injurious, and that it will never fail to improve the condition of the ulcer, and of the surrounding parts, reducing the enlargement, and removing the deformity of the toe; so that, even should the operation be ultimately necessary, the surgeon will be better enabled to ascertain the exact situation and extent of the disease, and thus, by a single operation, to remove the whole of the matrix so effectually, as to secure his patient from any relapse; therefore, the time which has been occupied in the fumigating process cannot be considered as lost or misapplied.—*Dub. Journ. of Med. Sci.*

